# A novel method of three-dimensional hetero-spectral correlation analysis for the fingerprint identification of humic acid functional groups for hexavalent chromium retention†

**DOI:** 10.1039/c7ra12146f

**Published:** 2018-01-19

**Authors:** Jia Zhang, Huilin Yin, Barnie Samuel, Fei Liu, Honghan Chen

**Affiliations:** Beijing Key Laboratory of Water Resources & Environmental Engineering, China University of Geosciences Beijing 100083 China feiliu@cugb.edu.cn

## Abstract

Two-dimensional hetero-spectral correlation analysis has been widely used for the interpretation of spectral changes of humic substances involved in various environmental processes. However, when three different types of spectroscopies are utilised, only a pairwise correlation can be achieved. In order to overcome this problem, a novel method of three-dimensional hetero-spectral correlation analysis with scaling technique was developed in this study, which can further establish a direct correlation between three different types of spectroscopies, including FTIR, ^13^C CP/MAS NMR, and XPS. The proposed method was applied to the fingerprint identification of undissolved humic acid functional groups for Cr(vi) retention, which is one of the most important points for understanding the migration and transformation of Cr(vi) in a subsurface environment. The results indicated that mainly free and dissociated carboxylic groups, phenolic groups, and polysaccharide participated in the reaction with Cr(vi), and these functional groups were mainly located at aromatic domains. Besides, the variations of functional groups were related to the reduction of Cr(vi), and the reduced Cr(iii) mainly bound to aromatic domains. The successful application of the proposed method demonstrated that it can serve as a promising tool for further investigations concerning more complicated environmental processes and even other scientific fields by supplying more detailed, reliable and visualised spectral information.

## Abstract 2

A method of three-dimensional hetero-spectral correlation analysis was developed for fingerprint identification of humic acid functional groups for Cr(vi) retention.

## Graphical abstract

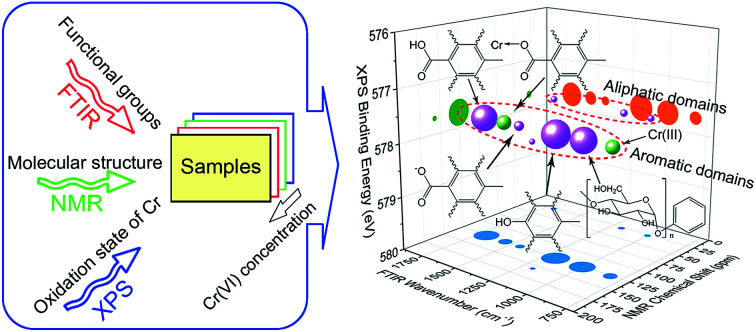

## 1. Introduction

Hexavalent chromium Cr(vi) is widely used for electroplating, leather tanning, and corrosion protection, and the infiltration of the chromium containing wastewater generated from the industrial processes has made it one of the most common heavy metal pollutants in contaminated soil and groundwater.^1^ Cr(vi) anions (mainly Cr_2_O_7_^2−^, HCrO_4_^−^, and CrO_4_^2−^) are very mobile in the terrestrial environment with extreme toxicity to organisms and potential carcinogenicity to humans, which make them of great scientific concern.^2^ Undissolved humic acid (HA) with a great quantity of active functional groups is widespread in the soil matrix, the content of which is generally two magnitudes higher than that of dissolved HA in soil.^3^ The undissolved HA has a significant retention effect on the migration of Cr(vi), and thus it is of great value to reveal the interaction mechanism between undissolved HA and Cr(vi) for understanding the fate of Cr(vi) in the subsurface.^4,5^ However, the reaction processes and HA molecular structures are extremely complex resulting in an unclear interaction mechanism, which may include the reduction of Cr(vi), the complexation of Cr(vi) anion or reduced Cr(iii) cation, and particularly the participation of a variety of functional groups that may be involved in the different reaction processes above.^6^ Thus the identification of the types of HA functional groups participating in the reaction with Cr(vi) is regarded as the most important step to reveal the interaction mechanism.

Infrared spectroscopy (IR) is considered as a useful tool to monitor the variation of HA functional groups during reaction with Cr(vi) due to its high sensitivity for organic functional groups.^7^ Spectral changes in mid-infrared region have been observed in previous investigations which were mainly assigned to the oxidation of carboxylic and phenolic groups and the formation of chelate between carboxyl and Cr ion.^8–10^ However, are there any other HA functional groups taking part in the reaction with Cr(vi)? What kinds of molecular structure domains are these functional groups more likely to be associated with? How can these spectral intensity changes indicating the variation of HA functional groups be directly linked to the interaction with Cr(vi)? At present, these questions still remain unclear, and the main reasons are as follows: firstly, for the presence of many similar functional groups in HA resulting from its complex molecular structure, the IR peaks overlap badly in mid-infrared region leading to significant decrease of the spectral resolution, and this makes it difficult to effectively identify the subtle intensity variation of the spectra artificially.^11^ Secondly, IR has been demonstrated to be effective for functional groups identification, but not for molecular structure, which is generally revealed by nuclear magnetic resonance (NMR). So how to cross-fertilize the two different classes of spectroscopy probes to provide more concrete information about the involving functional groups still remains to be solved. Thirdly, little attention has been given to the transformation of Cr valence states on the surface of undissolved HA in the past researches, and it was generally considered that all Cr(vi) removed from the liquid phase tended to react with HA functional groups.^12^ However, it has been found in our previous investigation that not all the Cr(vi) adsorbed onto HA is reduced to Cr(iii), and a fair amount of Cr still remains in hexavalent state on HA, which indicates that not all the Cr(vi) removed from liquid phase tend to induce the reaction with certain functional groups.^13,14^ Therefore, it is of great importance to make a direct association between the variation of functional groups and Cr(vi) transformation on HA that can be characterised by X-ray photoelectron spectroscopy (XPS).

Two-dimensional hetero-spectral correlation analysis, which integrates two different types of spectra obtained from a system under the same perturbation and using multiple spectroscopic probes, can serve as a promising tool to solve above problems.^15,16^ It can achieve hetero-correlation between completely different types of physical techniques, such as IR and NMR, to shed light on the interpretation of molecular functional groups and structure synchronous variation of samples under certain external perturbation.^17^ In recent years the 2D hetero-spectral correlation analysis has been widely used in the field of environmental science.^18–27^ However, in some situations more than two types of spectroscopies need to be used for the characterisation of the same experimental sample, and by this situation only a pairwise correlation can be achieved according to the current method system.^28^ Therefore, how to achieve a direct correlation analysis for three different types of spectroscopy is of great value for the hetero-spectral correlation analysis theory to supply more detailed, reliable and visualised spectral information.

In the present work, a method of three-dimensional hetero-spectral correlation analysis was proposed and verified, and then the novel method was employed for the investigation of the undissolved HA functional groups for Cr(vi) retention by achieving the three-dimensional hetero-spectral correlation among three different types of spectroscopies, namely FTIR, ^13^C CP/MAS NMR, and XPS, which respectively reflect the variation of functional groups, molecular structure, and Cr speciation on HA. The preliminary experiment was conducted under pH ranged from 1 to 5, and it was found that the lower pH was beneficial for the retention of Cr(vi) by HA. Therefore, in order to get the results within an acceptable time scale and make the spectra variation with a detectable extent, we mainly presented the results obtained at pH 1. This situation is equally common in contaminated sites associated with electroplating and leather tanning industries,^29,30^ where the extremely acidic wastewater (pH 1) discharged into the environment directly or by the means of seepage pits have been reported in many sites.^31^

## 2. Materials and methods

### 2.1 Sample preparation

Standard HA was purchased from Sinopharm Chemical Reagent Co., China, and was sieved through a 74 μm sieve to remove course particulates, and homogenised. The elemental composition, acidic groups content, ash content, water content, surface area, point of zero charge (PZC) can be found in our previous work.^13^

A series of 250 ml of Cr(vi) solutions with different initial concentrations ranging from 0.5 to 8 mM were added into 300 ml brown flasks containing 125.0 ± 0.5 mg undissolved HA. The solution contained a background electrolyte of 0.01 M NaCl, and the initial pH was adjusted to 1 by adding 2.5 M HCl. Each solution was shaken at 25 °C using a horizontal shaker with an intensity of agitation of 200 rpm for 20 d (the preliminary experiment proved that 20 d was sufficient for reaction equilibrium). All experiments were performed in triplicate. The vacuum filtration was used to separate undissolved HA from the solutions, and HA samples were freeze-dried for FTIR, ^13^C CP/MAS NMR and XPS analysis. The filtrate was filtered through a 0.22 μm membrane, and the concentration of Cr(vi) in the filtrate was determined using a UV/vis spectrophotometer (SHIMADZU UV-1800) at 540 nm after reaction with 1,5-diphenylcarbazide indicator (DPC).^32^ Total chromium was determined using ICP-AES (SPECBLUE) at 283.56 nm. The Cr(iii) concentration in the filtrate was determined by the difference between total chromium and Cr(vi) concentration.

### 2.2 FTIR, ^13^C CP/MAS NMR, and XPS characterization

FTIR spectra of samples were obtained on an IR spectrometer (Bruker LUMOS, Germany) at room temperature. All samples were fully ground to guarantee high homogeneity prior to tests. The samples were uniformly mixed with dried KBr powder at mass ratio of 1 : 200. Each spectrum was obtained after 64 scans with 2 cm^−1^ resolution, and the spectrum used for analysis was the average of scanning results obtained from triplicate samples.

Solid-state ^13^C CP/MAS NMR spectra of the samples were collected on a Bruker AVANCE III 400 NMR spectrometer with 4 mm NMR rotors with Kel-F caps. NMR spectra were obtained by applying the following parameters: rotor spin rate of 13 kHz, 1 s recycle time, 2 ms contact time, 20 ms acquisition time, and 10 000 scans. Chemical shifts were calibrated with adamantane.

XPS was measured with Thermo escalab 250XI. The X-ray excitation was provided by a monochromatic Al Kα (excitation energy 1486.6 eV). The binding energies of the spectra were corrected using the hydrocarbon component of adventitious carbon at 284.8 eV.

### 2.3 Two-dimensional and three-dimensional hetero-spectral correlation analysis

According to the theory of two-dimensional spectroscopy correlation analysis (2DCOS) proposed by Noda,^33^ the dynamic spectra which represent the variation of spectral intensity compare with reference spectrum can be calculated from the following:

1 *x̃*_*j*_(*v*) = *x̃*(*v*,*t*_*j*_) = *x*(*v*,*t*_*j*_) − *x̄*(*v*), *j* = 1,2,⋯, *m*

where the variable *v* represents the spectral index, such as the wavenumber of IR. *x̃*(*v*,*t*_*j*_) denotes the dynamic spectra measured at *m* equally spaced points in perturbation *t* between *t*_1_ and *t*_*m*_, and the average spectrum 
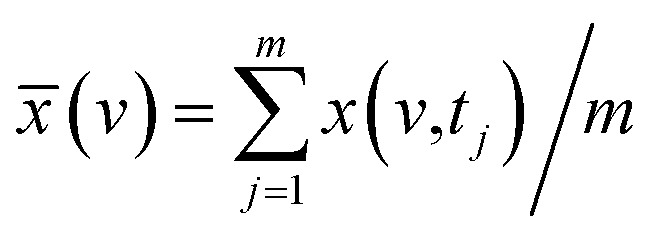
 has been subtracted from raw data *x*(*v*,*t*_*j*_). The synchronous correlation intensity for two-dimensional hetero-spectral correlation analysis can be directly calculated from the following:2
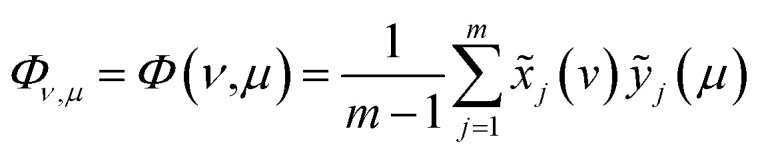
where the *v* and *μ* represent two different types of spectral indexes, such as the wavenumber of IR and chemical shift of NMR.

In order to achieve the correlation analysis for three different types of spectroscopy simultaneously, we first proposed a concept of three-dimensional hetero-spectral correlation intensity *Ω*_*ν*,*μ*,*λ*_, which can be calculated from the following:

3 *Ω*_*ν*,*μ*,*λ*_ = *Φ*_*ν*,*μ*_*Φ*_*ν*,*λ*_*Φ*_*μ*,*λ*_

As the discrepancies of variation extent for different spectral signals, during the calculation of correlation intensities, the signal variations with large amplitudes tend to dominate the correlation map and obscure the equally important details arising from relatively subtle but significant signal changes. This phenomenon has been reported previously,^26,27^ and Noda introduced scaling techniques to overcome this problem resulting in an desired effect.^34^ In this study we also employed similar method to solve the scale problem.

The standard deviation of spectral correlation intensity at *v* is defined as follows:4
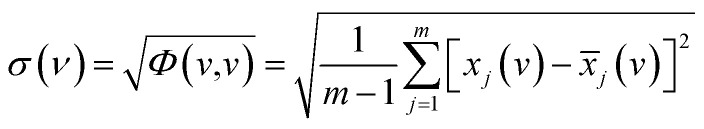


According to the scaling techniques suggested by Noda, the scaled three-dimensional hetero-spectral correlation intensity is given by:

5 *Ω*^(scaled)^_*ν*,*μ*,*λ*_ = *Φ*_*ν*,*μ*_*Φ*_*ν*,*λ*_*Φ*_*μ*,*λ*_[*σ*(*ν*)^2^*σ*(*μ*)^2^*σ*(*λ*)^2^]^−*α*^

where the *α* is the scaling factor limited from 0 to 1.0.

Prior to correlation analysis, the spectra of FTIR were normalized and denoised by Savitzky–Golay smoothing. The peak fitting of XPS data was conducted on XPSPEAK41 software. The 2DCOS analysis was produced using 2Dshige software (Kwansei-Gakuin University, Japan). A homemade software TDCOS 1.0 for three-dimensional hetero-spectral correlation analysis was developed using Visual Basic by one of the authors (Jia Zhang).

## 3. Results and discussion

### 3.1 Analysis of simulated data

#### Variation of simulated spectra

**Fig. 1:**
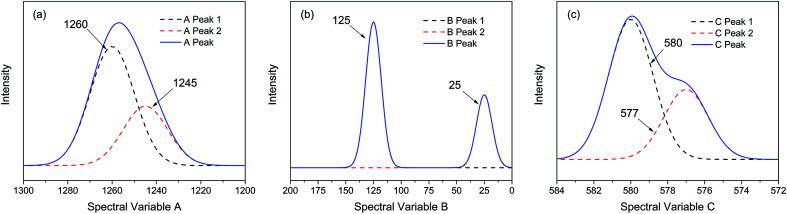
Three different types of simulated spectra with different overlapping characteristics. (a) Badly overlapped; (b) entirely isolated; (c) slightly overlapped.

In order to elucidate the features and reliability of three-dimensional hetero-spectral correlation analysis with scaling technique, the proposed method was applied to a series of simulated spectra. Three simulated spectra are involved in this section, namely A, B and C, which represent three different types of spectra obtained from a single experimental sample. Each trace of the spectra, as a function of different spectral variable, consists of two independent peaks with quite different overlapping characteristics as shown in Fig. 1. The three types of spectra are aimed to simulate the real spectral peaks under different overlapping conditions as well.

**Fig. 2:**
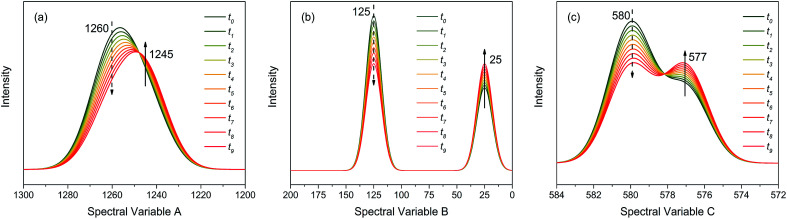
Three different types of simulated dynamic spectra with different variation directions and extend. (a) Badly overlapped; (b) entirely isolated; (c) slightly overlapped.

As shown in Fig. 2, the intensities of peaks in each spectrum vary in different directions and extent as a function of the external perturbation variable *t*, which can be any physical quantities that may influence the spectral intensities, such as time, temperature, and concentration. These independent peaks and intensity variations are given as a Gaussian peak function:^35^6
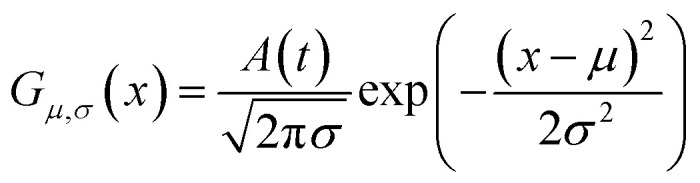
where *A*, *x*, *μ*, and *σ* are, respectively, the peak intensity coefficient, spectral variable, peak position, and full-width at half-height (FWHH). We assume that *A* only depends on *t*, and the parameters for the simulated spectra are summarised in Table 1.

**Table 1:** Parameters of Gaussian peak function for the simulated spectra

Peak	*A*(*t*)	*μ*	*σ*
A peak 1	1 − 1/20 × *t*	1260	10
A peak 2	1/2 × (1 + 1/20 × *t*)	1245	10
B peak 1	1 − 1/30 × *t*	125	7
B peak 2	1/2 × (1 + 1/30 × *t*)	25	7
C peak 1	1 − 1/30 × *t*	580	1.2
C peak 2	1/2 × (1 + 1/30 × *t*)	577	1.2

**Fig. 3:**
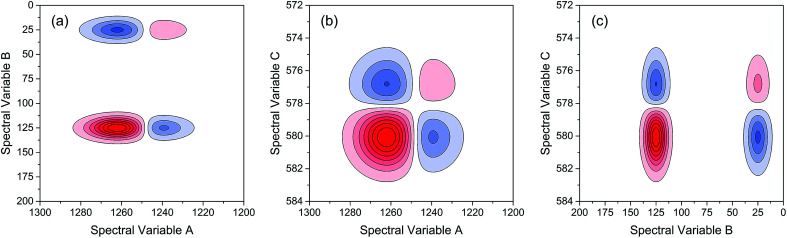
Two-dimensional hetero-spectral correlation maps of simulated data. (a) A and B; (b) A and C; (c) B and C. Red regions represent the positive correlation intensity; blue regions represent the negative correlation intensity.

As indicated, the intensities of peak 1 in all the three spectra decreased to a large extent, and that of peak 2 increased in a small extent. In order to clarify the correlation relationship between independent peaks of the three different types of spectra, a traditional two-dimensional hetero-spectral correlation was achieved in the form of pairwise correlation as shown in Fig. 3. According to the principles of hetero-spectral correlation analysis, the variation directions of corresponding peaks of two different types of spectra can be determined by the colour-filled regions in the maps, where the red and blue regions respectively indicated that the corresponding peaks varied simultaneously in the same and opposite directions, and the absolute value of the colour-filled region reflects the variation extent of corresponding peaks. For example, the red region with a relatively large positive value of correlation intensity at the bottom left corner of Fig. 3(a) represents that the spectral intensities at 1260 (spectra A) and 125 (spectra B) vary in the same direction with a relatively large extent, and the blue region with a relatively small negative value of correlation intensity at the bottom right corner of Fig. 3(b) represents that the spectral intensities at 1245 (spectra A) and 580 (spectra C) vary in the opposite direction with a relatively small extent. It can be seen that the two-dimensional hetero-spectral correlation analysis can serve as a useful tool to reveal the correlation relationship between the variation of two different types of spectra, however, obviously it is no longer a visualised way to show the correlation relationship between three different types of spectra on account of its failure to establish a direct correlation analysis between them.

#### Properties of three-dimensional hetero-spectral correlation analysis

**Fig. 4:**
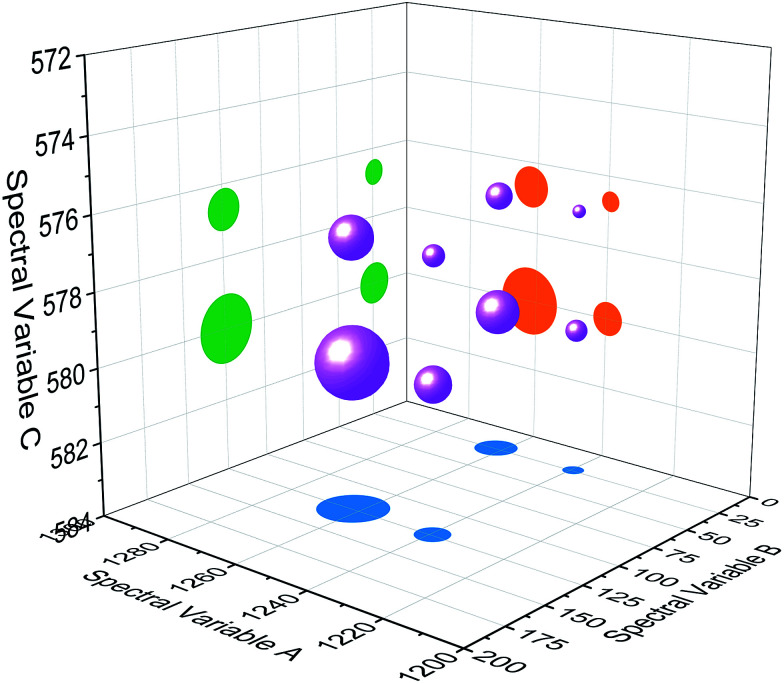
Bubble diagram of three-dimensional hetero-spectral correlation for simulated data. Scaling factor *α* = 0.75.

The three-dimensional hetero-spectral correlation analysis result was shown in a bubble diagram form (Fig. 4). The positions of the bubbles indicate that the corresponding spectral variables of the three different types of spectra have correlation relationship, and the diameters of the bubbles indicate the relative correlation intensities (with a scaling factor 0.75). It can be seen that the overlapped peaks are separated from each other effectively, and the largest and smallest bubbles respectively indicate the correlation of the most significant three decreasing peak changes (1260 of A, 125 of B, and 580 of C) and the less significant ones (1245 of A, 25 of B, and 577 of C) in the three sets of dynamic spectra as shown in Fig. 2.

**Fig. 5:**
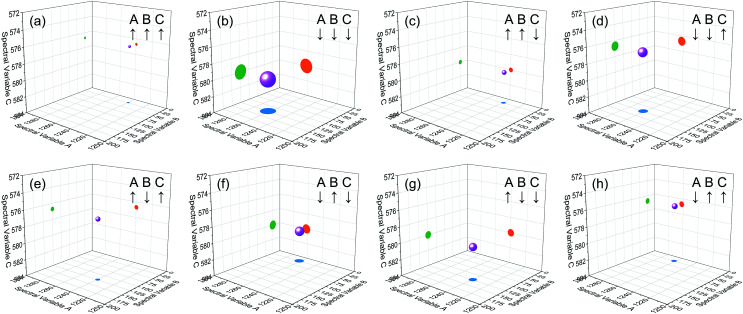
Bubble diagrams for eight different situations of peak changing characteristics from the three sets of simulated dynamic spectra. A, B and C represent the three different types of simulated dynamic spectra. ↑ indicates the peak intensity increases with external perturbation, and ↓ indicates the peak intensity decreases with external perturbation. Scaling factor *α* = 0.75.

Based on the theory of three-dimensional hetero-spectral correlation analysis, the correlation intensity can only be positive, while it is obvious that these bubbles indicate various combinations of peak changing directions determined by three different types of spectra. Consequently, it is of great value to differ the various situations of peak changing directions combination by dividing these bubbles into several groups (Fig. 5). As indicated, the bubbles in Fig. 4 were divided into eight situations, which respectively correspond to different combinations of peak change way obtained from the three different types of spectra including all the possible situations. The detailed change situations are marked in the figures, and the result of each situation can be obtained from the TDCOS 1.0 software automatically.

As shown above, three aspects of information can be obtained from the three-dimensional hetero-spectral correlation analysis: (1) which three different spectral variable from various spectroscopies have correlation properties; (2) how the relative correlation intensities are for these correlation bubbles; (3) what are the detailed changing directions of the three changing peaks obtained from the three sets of dynamic spectra for each correlation bubble. Compared with the traditional two-dimensional hetero-spectral correlation analysis, the proposed method has three aspects of advantages: (1) a direct correlation relationship can be established among three different types of spectra instead of a pairwise correlation; (2) the result of specific situation of peak changing direction combination can be obtained discretionarily using the homemade software; (3) the method supplies a more visualized, stabilized and reliable way to illustrate the correlation result of spectral intensity variation of three different types of spectra obtained from a single experimental sample under external perturbation.

### 3.2 Analysis of practical data

#### Adsorption of Cr(vi) by HA

The results of adsorption of various initial concentration of Cr(vi) by undissolved HA are shown in Fig. S1,† and it can be found that the amount of adsorbed Cr increased with the increasing initial concentration of Cr(vi). The result of desorption experiment (shown in Fig. S2†) indicated that the amount of reduced Cr(iii) increased with the increasing initial concentration of Cr(vi) as well, which means that with initial Cr(vi) concentration increasing more HA functional groups tend to be involved in the adsorption and reduction with Cr(vi).

#### FTIR, ^13^C CP/MAS NMR, and XPS analysis

**Fig. 6:**
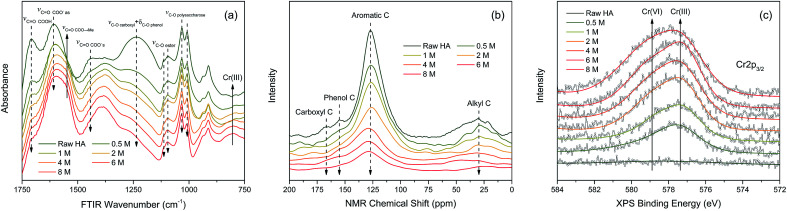
(a) FTIR, (b) ^13^C CP/MAS NMR, and (c) Cr 2p XPS spectra obtained from HA after reacting with different initial Cr(vi) concentrations ranged from 0.5 to 8.0 mM. The raw HA is referring to the HA without any perturbation.

A set of FTIR spectra were obtained to identify the HA functional groups variations after reacting with various initial concentration of Cr(vi) (Fig. 6(a)). As indicated, the intensities of absorption peaks at 1708, 1443, and 1236 cm^−1^ gradually decreased with the increasing Cr(vi) concentration, which were respectively attributed to the C=O stretching of free carboxyl,^36^ C=O stretching of dissociated carboxyl,^8^ and C–O stretching of phenol.^9^ The intensities of absorption peaks at 1113, 1095, 1032, 1008 cm^−1^ slightly decreased with the increasing Cr(vi) concentration, which were assigned to the C–O stretching of ester and polysaccharose.^37–39^ In contrast, the intensities of absorption peak at 1548 cm^−1^ increased significantly with increasing Cr(vi) concentration, which was ascribed to chelated carboxylic groups.^10^ The increased intensity of the peak at 804 cm^−1^ was very likely associated with Cr(iii) accumulation which was supported by the FTIR spectrum of CrCl_3_·6H_2_O (Fig. S3†).

In order to indicate the molecular structure variations of HA after reacting with Cr(vi), a set of NMR spectra were carried out and the result is shown in Fig. 6(b). The intensities of the peaks at 168, 155, 127, and 30 ppm decreased with the increasing Cr(vi) concentration, which were attributed to carboxyl carbons, phenol carbons, aromatic carbons, and aliphatic carbons respectively.^40^ Among them, the intensity decrease of aromatic carbons peak was the most significant one. The attenuation of NMR peak intensity in this study can be caused by two factors: (1) the corresponding carbons transformed into other types of carbons resulting from the oxidation by Cr(vi); (2) the chemical shift of corresponding carbons was strongly shielded owing to the diamagnetic effect of the Cr valence electrons.^41^

The oxidation state variation of Cr on HA was further determined by Cr 2p XPS, and the result is shown in Fig. 6(c). The peaks at 577.6 and 578.9 eV increased substantially with the increasing aqueous Cr(vi) concentration, which were attributed to Cr(iii) and Cr(vi) respectively.

**Fig. 7:**
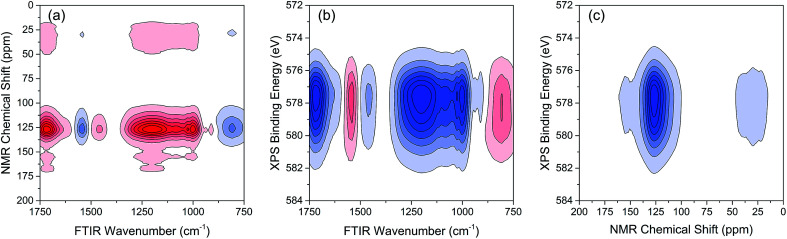
Two-dimensional hetero-spectral correlation between different types of spectra. (a) FTIR-NMR; (b) FTIR-XPS; (c) NMR-XPS. Red regions represent the positive correlation intensity; blue regions represent the negative correlation intensity.

The variations of HA functional groups and molecular structures were essentially induced by the reaction with Cr. Thus the spectral intensity variations of FTIR, NMR and XPS, which respectively reflect the characteristics of HA functional groups, molecular structures, and Cr oxidation state distributions, should have certain correlation relationships with each other. Therefore, the traditional two-dimensional hetero-spectral correlation analysis was utilised to verify the correlation relationship between different types of spectroscopy, and the results are shown in Fig. 7. It can be seen in Fig. 7(a) that the FTIR bands at 1708, 1443, 1236 and 1032 cm^−1^ were positively correlated with NMR band at 127 ppm, and the FTIR band at 1548 cm^−1^ was negatively correlated with NMR band at 127 ppm. This indicated that the HA functional groups participating in the reaction with Cr(vi) were mainly related to the aromatic domain of HA molecules. According to Fig. 7(b) and (c), the FITR and NMR bands were strongly correlated with XPS band at 577.6 eV that represents the signal of reduced Cr(iii), which indicated that the variation of functional groups were mainly induced by the reduction of Cr(vi). As mentioned above, when three types of spectra are employed for correlation analysis, the two-dimensional hetero-spectral correlation analysis is obviously no longer a visualised way to show the correlation relationship between them.

#### Three-dimensional hetero-spectral correlation analysis

**Fig. 8:**
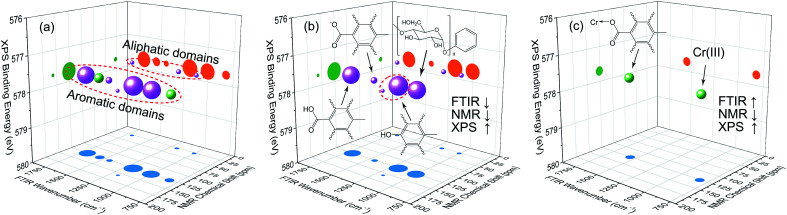
Bubble diagram of three-dimensional hetero-spectral correlation analysis for FTIR, NMR, and XPS. ↑ indicates the peak intensity increases with external perturbation, and ↓ indicates the peak intensity decreases with external perturbation. Scaling factor *α* = 0.75.

The results of three-dimensional hetero-spectral correlation analysis were shown in Fig. 8. It can be seen that all correlation bubbles were separated from each other entirely, and it is easier to point out the corresponding spectral bands of the correlation bubbles. As shown in Fig. 8(a), all correlation bubbles were located on the XPS plane of 577.6 eV that corresponds to reduced Cr(iii). This further confirms that the variations of HA molecular structures and functional groups are both directly related to the reduction of Cr(vi). Additionally, the correlation bubbles can be divided into two parts, which were respectively located on the NMR planes of 30 and 127 ppm, representing the variations of aliphatic and aromatic domains. The correlation intensities of aromatic carbons with other factors were much higher than that of aliphatic carbons, which indicated that the reduction of Cr(vi) mainly occurred at the aromatic domains of HA. The conclusion is consistent with that of two-dimensional hetero-spectral correlation analysis.

According to the results of three-dimensional hetero-spectral correlation analysis obtained from the homemade software, the correlation bubbles were divided into two groups with different peaks changing direction combinations. The correlation bubbles in Fig. 8(b) were involved in the situation that with increasing Cr(vi) concentration the peak intensities of FTIR and NMR decreased and that of XPS increased, which indicated that the corresponding functional groups and molecular structures participated in the reduction of Cr(vi) into Cr(iii). The functional groups for Cr(vi) reduction mainly included free carboxylic groups (1708 cm^−1^), dissociated carboxylic groups (1443 cm^−1^), phenolic groups (1236 cm^−1^), and polysaccharide (1032 cm^−1^), which were more likely located at aromatic domains (127 ppm). Meanwhile, the correlation bubbles in Fig. 8(c) were involved in the situation that with increasing Cr(vi) concentration the peak intensities of NMR decrease and that of FTIR and XPS both increased, which indicated that the reaction between HA and Cr(vi) resulted in the formation of corresponding complex of carboxyl-Cr (1548 cm^−1^). Additionally, the reduced Cr(iii) (804 cm^−1^) was more likely located at the aromatic domains of HA.

## 4. Conclusions

In this study, the novel method of three-dimensional hetero-spectral correlation analysis with scaling technique was first proposed to achieve a fingerprint identification of undissolved HA functional groups for Cr(vi) retention by establishing a direct correlation between three different types of spectroscopies, including FTIR, ^13^C CP/MAS NMR, and XPS. The results showed that mainly carboxyl, phenol, and polysaccharide were involved in the retention of Cr(vi) by HA, and all of them were more likely located at the aromatic domains of HA. Additionally, the variations of HA functional groups and molecular structure were both related to the reduction of Cr(vi), and the reduced Cr(iii) was mainly bound to aromatic domains. Compared with traditional two-dimensional hetero-spectral correlation analysis, the three-dimensional hetero-spectral correlation analysis can serve as a promising tool to supply much more detailed, reliable and visualized spectral information, and it is of great potential to extend it to other types of spectroscopies for further investigations concerning more complicated environmental processes and even other scientific fields.

## Conflicts of interest

The authors declare no conflict of interest.

## Supplementary Material

RA-008-C7RA12146F-s001
