# Supplementary material for: A novel method of three-dimensional hetero-spectral correlation analysis for the fingerprint identification of humic acid functional groups for hexavalent chromium retention
Source: RSC Adv. 2018 Jan 19;8(7):3522–9. doi: 10.1039/c7ra12146f (PMC9077723; doi:10.1039/c7ra12146f)
Supplement: RA-008-C7RA12146F-s001 [file RA-008-C7RA12146F-s001.pdf]

Electronic Supplementary Material (ESI) for RSC Advances.
This journal is © The Royal Society of Chemistry 2018

Supplementary data for:

A novel method of three-dimensional hetero-spectral correlation analysis for the fingerprint identification of humic acid functional groups for hexavalent chromium retention

Jia Zhang, Huilin Yin, Barnie Samuel, Fei Liu*, and Honghan Chen

Beijing Key Laboratory of Water Resources & Environmental Engineering, China University of Geosciences, Beijing 100083, China

——**Submit to *RSC Advances***

Corresponding author:

Fei Liu

Beijing Key laboratory of Water Resources and Environmental Engineering, China University of Geosciences, Beijing 100083, P. R. China

E-mail: feiliu@cugb.edu.cn

Summary:

8 pages

1 table

4 figures

Adsorption isotherm

The results of isothermal adsorption of Cr(VI) by undissolved HA are shown in Fig. S1. The adsorption quantity of Cr(VI) increased significantly with the increasing concentration of Cr(VI) in the solution, and parts of the adsorbed Cr(VI) were released into liquid phase after being reduced to Cr(III) resulting in the difference between adsorption quantity of Cr(VI) and Cr(T).

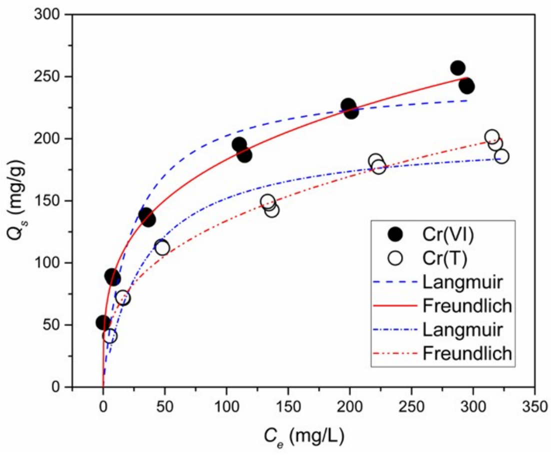

Fig. S1 Isothermal adsorption for various initial concentrations of Cr(VI) ranging from 0.5 to 8 mM onto 125 mg HA under pH 1 at 25 ℃ over 20 d. Cr(T) = total Cr.

The Langmuir and Freundlich isothermal models were utilized to fit the experiment results respectively. The Langmuir isotherm assumes that the adsorbent surface is structurally homogeneous and the binding sites have the same affinity for adsorbate, where only monolayer adsorption occurs without interactions between adsorbed molecules.[1] The Langmuir equation is given as:

$$Q_s = \frac{Q_m \times K_L \times C_e}{1 + K_L \times C_e} \tag{7}$$

where Q_s (mg/g) and Q_m (mg/g) represent the adsorption quantity and maximum

adsorption quantity on HA respectively; C_e (mg/L) is the equilibrium concentration of the adsorbate in the solution; K_L (L/mg) is the constant related to the affinity of the binding sites.

The empirical Freundlich isotherm assumes that the adsorbent surface is energetically heterogeneous, where the adsorbed molecules are interactive and the adsorption quantity raises infinitely with the increasing concentration of adsorbate in liquid phase.[2] The Freundlich equation is given as:

$$Q_s = K_F \times C_e^{\frac{1}{n}} \tag{8}$$

where Q_s (mg/g) represents the adsorption quantity on HA; C_e (mg/L) is the equilibrium concentration of the adsorbate in the liquid phase; K_F and n are the constants related to adsorption capacity and intensity respectively.

The fitting parameters for Langmuir and Freundlich isotherm are listed in Table S1. Compared with Langmuir isotherm model, Freundlich isotherm model fits better with the experimental data of both Cr(VI) and Cr(T) due to the higher correlation coefficients, which indicated that the HA surface was very heterogeneous. This is consistent with the uneven distribution of oxygen-containing functional groups with different reaction activities on HA surface as shown in the SEM-EDS mapping image for oxygen element (Fig. S4).

Table S1. Langmuir and Freundlich isotherm model parameters and R-squared values calculated from isothermal adsorption experimental data of Cr(VI) and Cr(T).

Model	Langmuir			Freundlich		
Parameter	K_L (L/mg)	Q_m (mg/g)	R^2	K_F	n	R^2

Cr(VI)	0.044	248.382	0.8594	49.457	3.516	0.9065
Cr(T)	0.030	202.639	0.9616	27.867	2.933	0.9866

In addition, the oxidation state distribution of Cr on HA was determined by a desorption experiment using 0.1 M Na_2HPO_4/KH_2PO_4 that is considered effective for the desorption of exchangeable Cr(VI),[3, 4] and result are shown in Fig. S2. As indicated, a fair amount of Cr still existed in the forms of adsorbed Cr(VI) on HA. Thus it is a question of value to be answered that whether the adsorbed Cr(VI) participating in the reaction with HA functional groups, and obviously this is difficult to be resolved by traditional analysis method.

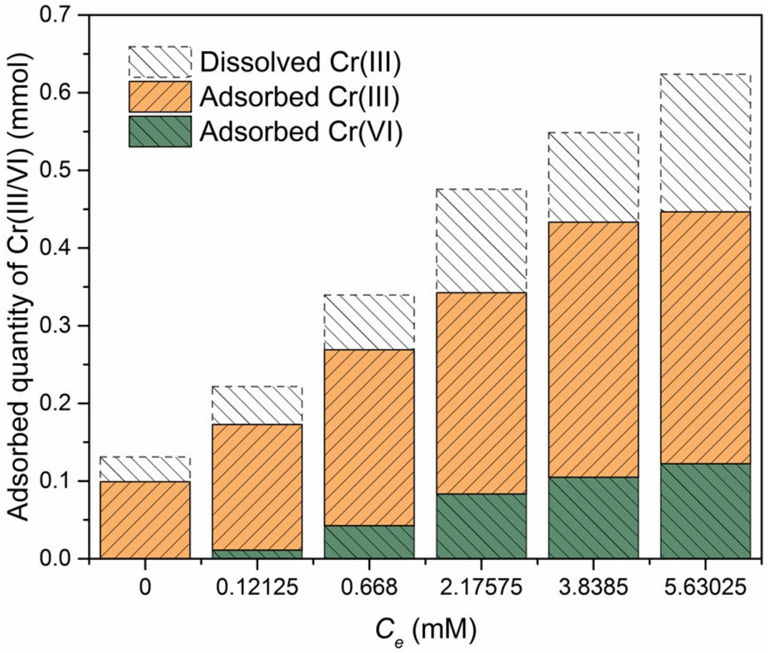

Fig. S2. Oxidation state distribution of chromium on HA under different initial Cr(VI) concentration ranging from 0.5 to 8 mM. The HA samples were desorbed by 250 mL 0.1 M NaOH for 1 d. The HA will be completely dissolved in the high concentration of NaOH, and the Cr(VI) was considered to be entirely released into aqueous phase, which corresponds to adsorbed Cr(VI).

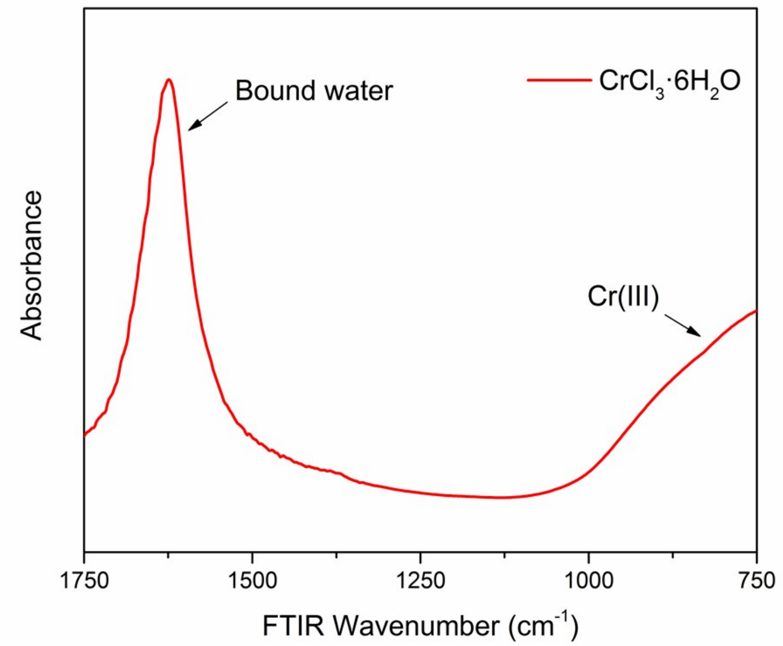

Fig. S3. FTIR spectrum of raw $CrCl_3 \cdot 6H_2O$.

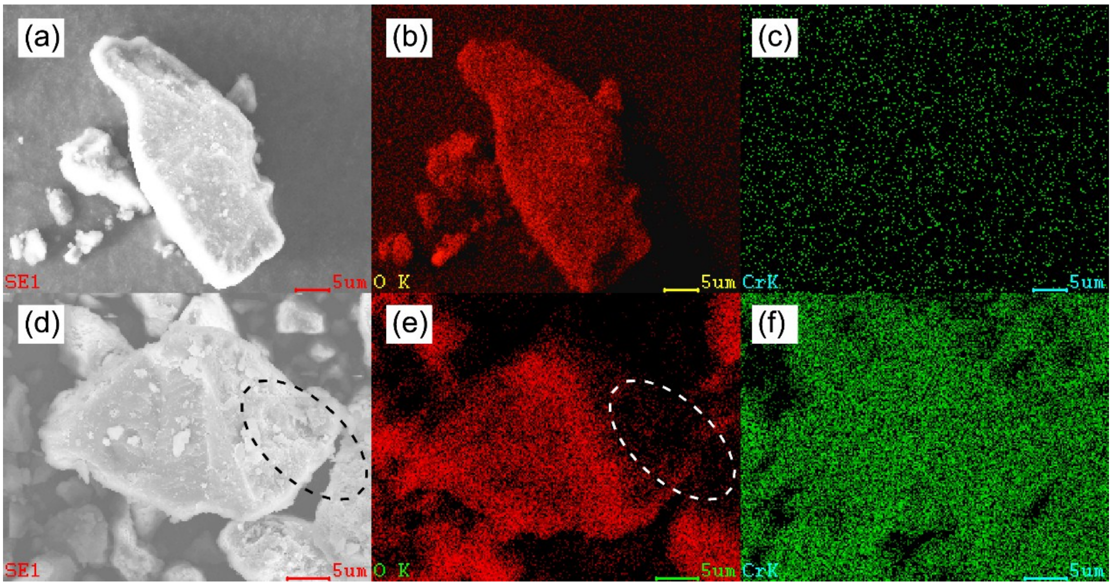

Fig. S4. SEM-EDS element mapping for HA before (a-c) and after (d-f) reacting with 8 mM Cr(VI). (a) and (d) are the SEM images; (b) and (e) are the oxygen element mapping images; (c) and (f) are the chromium element mapping images.

References

1. Y. Ren, H. A. Abbood, F. He, H. Peng and K. Huang, *Chem. Eng. J.*, 2013, **226**, 300–311.

2. Y. Chen and J. Wang, *Chem. Eng. J.*, 2011, **168**, 286-292.

3. P. Janos, V. Hula, P. Bradnova, V. Pilarova and J. Sedlbauer, *Chemosphere*, 2009, **75**, 732-738.

4. N. Kožuh, †, a. J. Štupar and B. Gorenc‡, *Environ. Sci. Technol.*, 2000, **34**, 112-119.
